# Mattering Matters: Examining the Moderating Role of Mattering in Cyberbullying Victimization-Outcome Relationships Among College Students

**DOI:** 10.3390/ijerph21111391

**Published:** 2024-10-22

**Authors:** Gary W. Giumetti, Robin M. Kowalski

**Affiliations:** 1 Department of Psychology, Quinnipiac University, Hamden, CT 06518, USA; gary.giumetti@quinnipiac.edu; 2 Department of Psychology, Clemson University, Clemson, SC 29634, USA

**Keywords:** college students, depression, cyberbullying victimization, life satisfaction, mattering, self-esteem

## Abstract

Recognized as a public health threat, cyberbullying victimization (CV), or bullying experienced through digital technologies, is mostly studied among adolescents, but evidence among college students suggests that it is prevalent and linked with negative outcomes. One protective factor that might reduce the impact of CV is mattering, which involves feeling significant and valued by others. In this study, we explored five hypotheses, including examining the moderating role of mattering in four CV-outcome relationships (self-esteem, life satisfaction, depression, and academic performance). Data were collected across two time-points from 134 college seniors using online surveys. Mattering moderated the CV-life satisfaction relationship and the CV-self-esteem relationship, such that, at low levels of mattering, there was no relationship, but, at average/high levels of mattering, there were negative CV-outcome relationships. To our knowledge, this is the first study to explore the role of mattering in relation to CV among college students and paves the way for additional research examining mattering as a moderator of other CV-outcome relationships. The fact that college students in the current study who did not feel that they mattered were seemingly unaffected by cyberbullying victimization highlights the need for both additional research and interventions, particularly with these individuals.

## 1. Introduction

Cyberbullying, or electronic bullying, refers to the use of digital technologies to bully others [1]. Whereas cyberbullying research has primarily examined adolescents [2], cyberbullying is also problematic among college students. In this group, prevalence rates for cyberbullying victimization (CV) range from 8 to 15% [3]. Recognized as a public health threat [4], cyberbullying is linked with several negative outcomes, including depression, anxiety, suicidal ideation, reduced academic performance, and lower self-esteem [5,6]. Previous research has examined protective factors that may moderate the victimization-outcome relationship, such as social support, personality [7], and coping strategies [8]. However, the protective role of psychological mattering in moderating the victimization-outcome relationship has not been examined. To extend this literature, the current study examined the moderating role of mattering in four CV-outcome relationships: self-esteem, life satisfaction, depression, and academic performance.

### 1.1. Theoretical Model

From a theoretical standpoint, we adopted the General Aggression Model (GAM) for CV [5] to support the current study. The GAM has been applied to other public health threats including domestic violence [9], pain [10], and global climate change [11]. This model positions person and situation factors as important precursors to cyberbullying encounters and the impact that such behavior might have on internal states, including cognition, affect, and arousal. For example, if an individual experiences CV, this may activate internal states such as negative affect (mood) as well as other feelings and thoughts related to belongingness with the perpetrator. After these internal states are activated, the GAM for CV predicts that individuals will engage in appraisal and decision-making processes, and may experience a variety of distal outcomes, such as decreased psychological and physical health, poorer social functioning, and increased behavioral problems [5]. Thus, this model suggests that the relationship between cyberbullying encounters and distal outcomes depends, in part, on the extent to which CV activates affect-based internal states. That is, the GAM for CV suggests that affect-based variables may serve as moderators of the CV-outcome relationships.

### 1.2. Present Study: Hypothesis Development

#### 1.2.1. Linking Cyberbullying Victimization to Negative Outcomes

For the current study, we aimed to replicate and extend previous research that has examined the relationship of CV with negative outcomes among college students. This includes studies that have found links between CV and decreased self-esteem (or perceptions of self-worth) [12,13], decreased life satisfaction (or feelings of happiness or contentment with one’s life) [14], and decreased academic performance (or one’s grades in college) [15]. Additionally, several studies have linked experiences of CV with increases in depressive symptoms (or feelings of sadness, loss of interest in activities, and low energy) among college students [12,16]. However, these studies have all taken a cross-sectional approach (that is, measuring CV and outcomes at only one time-point) to examine these relationships, and thus are limited in their ability to make claims about directionality and causation. We are aware of only a few studies that have taken a longitudinal approach to examine the relationships between CV and negative outcomes among college students [17,18]. We extend this previous research by examining the links among CV and self-esteem, life satisfaction, academic performance and depressive symptoms using a multi-wave design among a college student sample from the USA. We propose the following hypotheses:

1. Experiences of CV in the Fall semester will be negatively related to levels of self-esteem in the Spring semester.
2. Experiences of CV in the Fall semester will be negatively related to life satisfaction in the Spring semester.
3. Experiences of CV in the Fall semester will be negatively related to academic performance in the Spring semester.
4. Experiences of CV in the Fall semester will be positively related to levels of depressive symptoms in the Spring semester.

#### 1.2.2. The Moderating Role of Mattering

In addition to examining the links between CV and negative outcomes, we also aimed to explore the moderating role of mattering in these relationships. Mattering deals with the feeling that others can depend on us, regard us as important, and are paying attention to us [19,20,21]. High levels of mattering are protective and linked with positive outcomes, such as life satisfaction [22]. For example, among dating violence victims, higher perceptions of mattering were linked with lower depression and better academic performance [23]. Individuals who think they do not matter feel insignificant and of low value to others. Such feelings are linked to negative personal [24], relationship [25], and societal outcomes [26].

In line with the GAM for CV, mattering might be considered an affect-based component of one’s present internal state that may impact the extent to which a victim experiences negative outcomes [5]. This is consistent with theorizing by Flett [26] regarding the role of mattering in moderating the relationship between traditional bullying and negative outcomes. More specifically, Flett predicted that bullying victimization experiences will be magnified for those who feel they do not matter.

Many negative outcomes associated with not mattering mirror those following CV. Whereas existing research has linked mattering with increased academic achievement [27], life satisfaction [28], and self-esteem [29], as well as lower levels of depression [30] to the best of the authors’ knowledge, no research to date has examined the relationship between mattering and CV. One study, however, found peer rejection, which one would expect to be related to perceptions of low mattering, to moderate the relationship between CV and peer aggression [31]. Based on the GAM for CV [5] and existing research on mattering and CV, we propose the following hypothesis:

1. Perceptions of mattering will moderate the relationships between CV and (a) self-esteem, (b) life satisfaction, (c) academic performance, and (d) depressive symptoms such that individuals who have higher perceptions of mattering will be less negatively impacted by CV.

## 2. Materials and Methods

### 2.1. Participants

Participants for the current study were recruited from the fourth-year undergraduates (seniors) of two universities in eastern USA. One university was a private university in the Northeast. The second was a large, public university in the Southeast. We received 523 responses at T1 (71% female; 82% White) and 390 responses at T2 (76% female; 85% White). Our final data set consisted of 134 respondents (25.6% retention rate; 80% female; 84% White, 5% Hispanic, 5% Asian or Asian American, 2% African American; 4% other; Mean age = 21.54, SD age = 4.67, age range: 19 to 65) who participated at both T1 and T2 (*N* = 37 from the private university and *N* = 97 from the large public university). We checked for differences between the two samples on the six study variables using a series of independent sample *t*-tests and found no significant differences (all *p*-values > 0.12). Therefore, we combined both samples for the analyses.

### 2.2. Procedure

The study was approved by the Institutional Review Boards at both universities. Because most of the existing research on cyberbullying victimization is cross-sectional in nature, a longitudinal design was used in the current study to examine mattering as a moderator and the predictive effects of cyberbullying victimization on outcomes 6 months later. To accomplish this, a link to a SurveyMonkey survey was sent via university email to all seniors at two universities during Fall 2021 (T1) and Spring 2022 (T2), approximately six months later. Participants were provided with a unique code which they entered with their survey responses. This allowed the researchers to link their data from T1 and T2. To incentivize participation, participants were told that they would be entered into a drawing for one of five $50 Amazon gift cards if they completed the survey. Participants first read the informed consent and agreed to participate. Then, they completed six self-report measures in counterbalanced order at both T1 and T2 (see below for the list of measures). After completing the survey, participants were thanked for their time, debriefed, and told that they would be contacted in six months to complete the survey again.

### 2.3. Measures

Participants completed the six self-report measures below (as part of a larger survey). Where appropriate, reverse-scoring was used (e.g., “At times, I think I’m no good at all”), with higher numbers indicating more of the construct of interest.

***Cybervictimization.*** To measure the frequency with which respondents had experienced cyberbullying victimization, the 17-item Workplace Cyberbullying Measure [32] was used, adapted to the school context. This measure has been previously adapted and used to measure cyberbullying in college student samples successfully in the past [17]. Using a 5-point scale (1 = *never*; 5 = *daily*), respondents indicated how often they had experienced each of the behaviors electronically through technology in the previous 6 months [e.g., “Received aggressively worded messages (e.g., using all capital letters, bold font or multiple exclamation marks)”]. Cronbach’s alpha = 0.89.

***Mattering.*** Psychological mattering or the extent to which participants felt they were important or significant to others was assessed with the General Mattering Scale [20]. Participants responded to each of the items using a 5-point scale (1 = *not at all*; 5 = *a lot*). A sample item was “How important do you feel you are to other people?”. Cronbach’s alpha = 0.82.

***Self-esteem*.** Self-esteem, or participants’ positive or negative evaluation of themselves, was measured using the 10-item Rosenberg Self-Esteem Scale [33]. Participants responded to each of the items using a 5-point scale (1 = *strongly disagree*; 5 = *strongly agree*). A sample item is “I am able to do things as well as most other people” (alpha = 0.89).

***Life satisfaction*.** Life satisfaction (or one’s positive evaluation of overall life quality) was measured with Diener et al.’s [34] 5-item Satisfaction with Life Scale (e.g., “So far I have gotten the important things I want in life”). Participants indicated the extent to which each of the items applied to them over the previous 6 months using a 5-point scale (1 = *strongly disagree*; 5 = *strongly agree*) (alpha = 0.88).

***Depression.*** Depression was measured with 7-items from the Depression, Anxiety, and Stress Scales [35]. A sample item is “I found it difficult to work up the initiative to do things”. Respondents indicated the extent to which each item applied to them over the previous 6 months using a 4-point scale (1 = *does not apply to me*; 4 = *applies to me very much or most of the time*). Cronbach’s alpha = 0.87.

***Academic performance.*** To determine overall academic performance, participants self-reported their grade point average (GPA), which ranges from 0 to 4.0 for the current semester. Meta-analytic evidence suggests that self-reported GPA is quite accurate, as Kuncel et al. [36] found a meta-analytic correlation of 0.90 between self-reported GPA and objective college GPA.

### 2.4. Data Analysis

Frequencies and Pearson’s correlations were conducted to gather descriptive data and examine the relationships between study variables (i.e., to test hypotheses 1–4). To analyze the moderating role of mattering in these relationships (hypothesis 5), we used the Process macro v4.1 [37], with T1 CV as the predictor, T1 mattering as the moderator, and T2 depressive symptoms, academic performance, life satisfaction, and self-esteem as outcomes in separate regression analyses. We mean centered both CV and mattering for each interaction to improve interpretation of regression coefficients and we used 5000 bootstrap samples to obtain confidence intervals around the regression coefficients.

## 3. Results

A large percentage of respondents reported having experienced at least one instance of CV “now and then”. Specifically, 90.3% reported CV at T1 and 93.3% at T2. However, less than 15% of participants at T1 and T2 indicated that they had experienced at least one instance of CV “at least monthly” (this may explain the low mean for CV, 1.53).

Descriptive statistics and intercorrelations can be viewed in Table 1. As seen in Table 1, the correlational results indicate that T1 CV was significantly positively related to T2 depressive symptoms (*r* = 0.39) and significantly negatively related to T2 life satisfaction (*r* = −0.29) and T2 self-esteem (*r* = −0.30), but not significantly related to T2 academic performance (*r* = −0.05). These findings provide support hypotheses 1, 2, and 4, but fail to support hypothesis 3.

Results of the moderation analyses are presented in Table 2. For hypothesis 5a, results indicated that CV, mattering, and the CV-mattering interaction all significantly predicted self-esteem. The CV-mattering interaction term explained an additional 2.3% of the variance in self-esteem. The results were similar for life satisfaction, with CV, mattering, and the CV-mattering interaction all significantly predicting life satisfaction. The CV-mattering interaction term explained an additional 2.8% of the variance in life satisfaction. Thus, hypothesis 5b was supported.

For hypothesis 5c, the results indicated that there were no significant predictors of academic performance (all *p*’s > 0.41), thus hypothesis 5c was not supported. For hypothesis 5d, both CV and mattering predicted depressive symptoms, but the CV-mattering interaction did not significantly predict depressive symptoms. Thus, hypothesis 5d was not supported.

**Figure 1:**
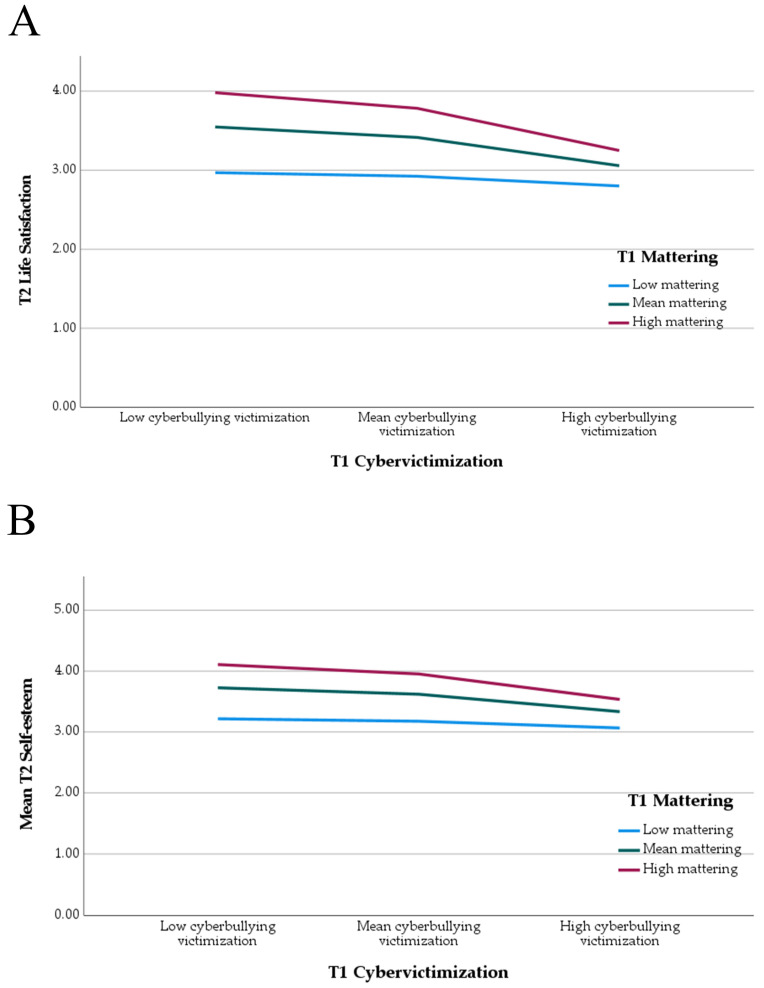
(**A**) graphical representation of the interaction between T1 CV and T1 mattering predicting T2 life satisfaction (see panel (**A**) above) and T2 self-esteem (see panel (**B**) above).

To understand the form of the significant interactions, we plotted simple slopes for the relationships between CV and self-esteem/life satisfaction at high (+1 SD), medium (at the mean), and low (−1 SD) levels of mattering (see Figure 1). The patterns are similar for both interactions—for low mattering, there is no significant relationship between CV and self-esteem/life satisfaction. However, at the mean and above the mean of mattering, there are significant negative relationships between CV and both self-esteem and life satisfaction.

## 4. Discussion

Given the prevalence of cyberbullying among college students and the fact that cyberbullying has been recognized as a public health threat [4], understanding the mental health outcomes associated with victimization as well as protective factors in this relationship is critical. Toward this end, we examined the longitudinal relationship between CV and four outcomes and the degree to which mattering might serve as a protective factor in these relationships. As an extension of previous longitudinal research among college students [17,38], we found links between CV experienced in the Fall semester and self-esteem, life satisfaction, and depressive symptoms in the Spring semester, supporting hypotheses 1, 2, and 4. However, we did not find a relationship between CV and academic performance (Hypothesis 3). These findings extend existing research among college students, which has primarily examined predictors of cyberbullying perpetration [39], and support meta-analytic findings across other longitudinal studies [6]. Cybervictimization experienced at T1 was associated with lower self-esteem and life satisfaction, as well as increased feelings of depression at T2. These findings are consistent with previous research with adolescents showing that CV is a risk factor for internalizing symptoms, such as anxiety and depression [6,40]. The failure to find a relationship between CV at T1 and academic performance at T2 may have stemmed from a ceiling effect of GPA, thus little variability. The mean GPA score of 3.54 suggests that most students were performing well in school.

With regard to the role of mattering, existing theoretical work suggested that the experience of CV would be intensified for individuals low in mattering [26]. This would be consistent with research showing the role of peer rejection as a moderator between cyberbullying victimization at T1 and cyberaggression at T2 [31]. However, our results suggest that individuals high in mattering may be more susceptible to the negative impact of CV. More specifically, for individuals with an average or above average level of mattering, life satisfaction and self-esteem decreased as CV increased. Individuals who felt they did not matter appeared unaffected by CV, as life satisfaction/self-esteem scores remained low and relatively steady as CV increased. We also found that mattering did not play a moderating role in the relationships between CV and depressive symptoms or academic performance. From the perspective of the GAM [5], mattering may function as a psychological state that is activated when a cyberbullying encounter occurs, forcing the victim to evaluate their relationship with the aggressor. If they perceive they do not matter, then they may not be as affected by the cyberbullying victimization. However, if they feel they matter a great deal, they may be surprised by the victimization and thus experience more negative outcomes. These findings extend the literature on mattering by being the first study (to our knowledge) to explore the moderating role of mattering in cyberbullying victimization-outcome relationships.

### 4.1. Practical Implications

The association of CV at T1 with lower self-esteem and life satisfaction and increased depressive symptoms highlights the need for prevention and intervention programs. The prevention programs should be designed to reduce the prevalence of CV whereas the intervention programs should be targeted toward victims to reduce the effects of the negative outcomes associated with their victimization.

One way to do this would be the development of programs and interventions tailored toward making people feel that they matter. For example, the National Suicide Prevention Lifeline developed “You Matter” as a forum for promoting mental health among youth. Additionally, Gibson et al. [41] utilized an intervention with African-American boys and found improvements in behavior and social-emotional skills. Similar programs can and have been developed in schools and should be developed in universities and workplaces.

The finding that individuals who did not feel that they mattered were unaffected by CV is disconcerting in that it suggests that (a) these individuals either expect others to treat them poorly and/or (b) they have given up. Previous research showing a link between anti-mattering and depression [42] and suicidality [24] highlights why attending to these unaffected people and intervening on their behalf is critical.

### 4.2. Future Research Directions and Limitations

As Flett [20] highlighted, people who do not feel like they matter feel insignificant. As such, these individuals might not be surprised by experiences of victimization; thus, these experiences may not impair their views of the self or their emotional reactions. Future research should examine whether individuals who are low in mattering have a history of victimization, as this may be one cause for low levels of mattering. Additionally, researchers should explore mattering and victimization over time to see if victimization erodes mattering and leads to future perpetration [43]. Existing research suggests that mattering is protective against depressive symptoms over time for adult women, but not men, among a Canadian sample [44]. Further research is needed to explore the links among mattering, self-esteem, life satisfaction, and depression over longer periods of time (or more than two measurement occasions) and in additional regions of the world to further understand how mattering changes over time and its links with important internalizing and externalizing variables. Future research should also examine the relationship between CV and mattering in light of demographic variables such as gender and socioeconomic status.

Our findings should be interpreted in light of several limitations. First, our dataset consisted of self-reported responses, so response sets and social desirability bias are possible. Whereas these concerns are lessened by our measurement of variables across two time-points with different response scales for each measure, future research that gathers data from sources beyond self-report is needed. Our sample is also limited in size, as only 134 respondents completed the survey at both time points, the majority of whom were female and White. Thus, our data may not represent young adults in the US more broadly. With regard to the sample size, however, a post-hoc power analysis indicated that we achieved a power of 0.97 or higher to detect the relationships between cyberbullying victimization and self-esteem, life satisfaction, and depressive symptoms.

## 5. Conclusions

The current study extends previous research examining the link between cyberbullying victimization and problematic outcomes. In addition, however, the study examines the moderating role of psychological mattering, or the extent to which people feel they are important or significant. Of concern, college students in the current study who did not feel that they mattered were seemingly unaffected by cyberbullying victimization, highlighting the need for both additional research and interventions, particularly with these individuals.

## Figures and Tables

**Table 1:** Descriptive Statistics and Intercorrelations Among Study Variables at Time 1 and Time 2.

#	Variable	M	SD	1	2	3	4	5	6	7	8	9	10	11
1	T1 CV	1.53	0.47											
2	T2 CV	1.52	0.48	0.72 *										
3	T1 Mattering	2.88	0.65	−0.09	−0.03									
4	T2 Mattering	2.93	0.68	−0.02	0.01	0.72 *								
5	T1 Depressive Symptoms	1.82	0.72	0.37 *	0.31 *	−0.37 *	−0.37 *							
6	T2 Depressive Symptoms	1.76	0.73	0.39 *	0.40 *	−0.35 *	−0.38 *	0.65 *						
7	T1 Life Satisfaction	3.15	0.81	−0.34 *	−0.30 *	0.39 *	0.43 *	−0.62 *	−0.45 *					
8	T2 Life Satisfaction	3.27	0.88	−0.29 *	−0.31 *	0.42 *	0.45 *	−0.60 *	−0.58 *	0.71 *				
9	T1 Self−Esteem	3.44	0.72	−0.33 *	−0.27 *	0.51 *	0.49 *	−0.74 *	−0.57 *	0.65 *	0.62 *			
10	T2 Self−Esteem	3.50	0.70	−0.30 *	−0.28 *	0.48 *	0.52 *	−0.67 *	−0.69 *	0.58 *	0.69 *	0.80 *		
11	T1 GPA	3.62	0.33	−0.04	−0.10	0.09	0.15	−0.03	−0.05	0.08	0.12	0.12	0.20 *	
12	T2 GPA	3.62	0.34	−0.05	−0.08	0.08	0.09	0.02	−0.08	0.07	0.11	0.08	0.16	0.74 *

*Note*. * *p* < 0.05. T1 = time 1 (Fall 2021); T2 = time 2 (Spring 2022); M = mean; SD = standard deviation; GPA = grade point average.

**Table 2:** Results from Moderated Regression Analyses to Test Hypotheses 5a–d.

Predictors	Outcome	ΔR2	B	SE	t	*p*
T1 CV	Self-Esteem	0.295, *p* < 0.001	−0.41	0.11	−3.78	<0.001
T1 Mattering			0.49	0.08	6.21	<0.001
T1 CV*T1 Mattering		0.023, *p* = 0.037	−0.35	0.17	−2.10	0.038
T1 CV	Life Satisfaction	0.239, *p* < 0.001	−0.50	0.14	−3.55	<0.001
T1 Mattering			0.54	0.10	5.23	<0.001
T1 CV*T1 Mattering		0.028, *p* = 0.033	−0.47	0.21	−2.21	0.03
T1 CV	Academic Performance	0.007, *p* = 0.662	−0.03	0.06	−0.40	0.690
T1 Mattering			0.04	0.05	0.83	0.411
T1 CV*T1 Mattering		0.001, *p* = 0.864	−0.02	0.10	−0.24	0.813
T1 CV	Depressive Symptoms	0.254, *p* < 0.001	0.57	0.12	4.86	<0.001
T1 Mattering			−0.35	0.09	−4.13	<0.001
T1 CV*T1 Mattering		0.004, *p* = 0.346	0.15	0.18	0.82	0.414

*Note.* B = unstandardized regression coefficient; SE = standard error; T1 = time 1 (Fall 2021); T2 = time 2 (Spring 2022).

## Data Availability

The data for this study can be viewed and downloaded from the following website: https://osf.io/p5vmz/?view_only=253bd57c24454824b3a5e95671a934f3 (accessed on 10 September 2024).
